# Effect of spaceflight experience on human brain structure, microstructure, and function: systematic review of neuroimaging studies

**DOI:** 10.1007/s11682-024-00894-7

**Published:** 2024-05-22

**Authors:** Sahar Rezaei, Homa Seyedmirzaei, Esmaeil Gharepapagh, Fateme Mohagheghfard, Zahra Hasankhani, Mahsa Karbasi, Sahar Delavari, Mohammad Hadi Aarabi

**Affiliations:** 1 Clinical Research Development Unit of Tabriz Valiasr Hospital, Tabriz University of Medical Sciences, Tabriz, Iran; 2 Department of Nuclear Medicine, Medical School, Tabriz University of Medical Sciences, Tabriz, Iran; 3 Sports Medicine Research Center, Neuroscience Institute, Tehran University of Medical Sciences, Tehran, Iran; 4 Department of para Medicine, Medical School, Tabriz University of Medical Sciences, Tabriz, Iran; 5 Department of radiology, Medical School, Tabriz University of Medical Sciences, Tabriz, Iran; 6 Institute for the Developing Mind, Children’s Hospital Los Angeles, Keck School of Medicine at the University of Southern California, Los Angeles, CA USA; 7 Padova Neuroscience Center (PNC), University of Padova, Padova, Italy; 8 Department of Neuroscience, University of Padova, Padova, Italy

**Keywords:** Spaceflight, Astronaut, Microgravity, MRI, Neuroimaging

## Abstract

Spaceflight-induced brain changes have been commonly reported in astronauts. The role of microgravity in the alteration of the brain structure, microstructure, and function can be tested with magnetic resonance imaging (MRI) techniques. Here, we aim to provide a comprehensive overview of Spaceflight studies exploring the potential role of brain alterations identified by MRI in astronauts. We conducted a search on PubMed, Web of Science, and Scopus to find neuroimaging correlates of spaceflight experience using MRI. A total of 20 studies (structural MRI n = 8, diffusion-based MRI n = 2, functional MRI n = 1, structural MRI and diffusion-weighted MRI n = 6, structural MRI and functional MRI n = 3) met our inclusion criteria. Overall, the studies showed that regardless of the MRI techniques, mission duration significantly impacts the human brain, prompting the inclusion of various brain regions as features in the analyses. After spaceflight, notable alterations were also observed in the superior occipital gyrus and the precentral gyrus which show alterations in connectivity and activation during spaceflight. The results provided highlight the alterations in brain structure after spaceflight, the unique patterns of brain remodeling, the challenges in drawing unified conclusions, and the impact of microgravity on intracranial cerebrospinal fluid volume.

## Introduction

Due to challenges such as increased radiation exposure, the effects of microgravity, and the potential psychological strains of social isolation and confinement, spaceflight presents a myriad of health risks (Clément, 2011). Prolonged space travel can have detrimental impacts on various physiological systems within the human body (Clément et al., 2020). For instance, upon entering a microgravity environment, individuals experience a cephalic fluid shift due to the loss of hydrostatic pressure in the caudocranial fluid columns (Nelson et al., 2014). This fluid redistribution has been linked to alterations in ocular structures and visual acuity, leading to a condition termed Spaceflight-Associated Neuro-ocular Syndrome (SANS) (Lee et al., 2018). Symptoms of SANS include global flattening of the eye, choroidal folds, optic disk edema, and a shift towards hyperopia, all of which carry the potential risk of causing permanent vision impairment (Macias et al., 2020). However, the exact physiological and pathological changes of the brain during spaceflights remain elusive.

In the brain, spaceflight has been consistently shown to induce morphological changes. For example, following long-duration missions, astronauts have exhibited alterations in gray matter volume (Koppelmans et al., 2016), the structures of ventricular walls (Van Ombergen et al., 2019), and the distribution of subarachnoid cerebrospinal fluid (CSF) (Van Ombergen et al., 2018). All these findings have been achieved through conventional structural magnetic resonance imaging (MRI). Structural MRI can quantify the volume and shape of various brain regions and has been long used in clinical settings (Zeinali et al., 2017). However, in research settings, the use of more advanced imaging modalities can complement these findings. In this regard, diffusion-based MRI holds great potential to reveal microstructural changes, especially in the white matter (Nabizadeh & Jameie, 2022). Examples include diffusion-weighted imaging (DWI) and diffusion tensor imaging (DTI), assessing the diffusion characteristics of water molecules by quantitative measures, e.g., fractional anisotropy (Moghaddam et al., 2024). Moreover, as there are reports of altered function of the brain after spaceflights (Burles & Iaria, 2023), functional MRI (fMRI) serves as an appropriate tool to find more details on spaceflight effects.

In addition to the exact structural and functional changes of the brain, the correlates of these changes remain to be elucidated. For example, while some astronauts embark on space travel for the first time, others have multiple missions under their belts with varying intervals between flights. Mission durations can also vary substantially, from as short as two weeks to as long as a year. However, it remains controversial whether the structural brain changes and intracranial fluid shifts are influenced by prior flight experiences. As we edge closer to multi-year human missions, such as those planned for Mars, understanding the potential influence of cumulative spaceflight experience on structural brain alterations becomes paramount. One area of investigation centers on the persistence of these changes during extended microgravity exposure. A comparative study between short-duration (one-month) missions and longer ones (lasting six months or more) has reported no significant relationship between flight duration and the degree of brain changes (Riascos et al., 2019). Meanwhile, another study has shown that longer missions on the International Space Station (ISS) induce greater fluid shifts as mission duration was associated with increases in right lateral and third ventricle volumes (McGregor et al., 2023). These inconsistent findings reinforce the idea that prolonged exposure to microgravity might affect the brain structure, necessitating more investigations.

It’s important to highlight that the evaluation of spaceflight’s impact on brain structure, microstructure, and function varies significantly across different studies. Given this heterogeneity, a comprehensive review is pivotal to reconcile these disparities and offer a clearer understanding by juxtaposing the diverse influences. A previous narrative review by (Roy-O’Reilly et al., 2021) in 2021 indicated that various brain regions undergo macrostructural changes during spaceflights. However, as the studies on spaceflight effects are being published at a rising speed, an updated, systematic review is needed to aggregate the available data. In this vein, our study collates and summarizes the findings from various spaceflight research that employed MRI data to investigate neural alterations in astronauts. In contrast to the mentioned review, we will be focusing only on the real-world spaceflight effects rather than terrestrial analogs, animal studies, and mere impacts on the clinical profile of the astronauts. Our primary objective is to discern the combined predictive power of MRI in gauging spaceflight-induced changes in the brain, either by comparing pre-flight and post-flight profiles or recruiting controls.

## Method

Our literature search was conducted using PubMed, Web of Science, and Scopus to identify all relevant studies published from January 2018 to July 2023. Keywords were (“Space flight” OR “spaceflight” OR “microgravity” OR “weightlessness” OR “spacecraft”) AND (“neuroimaging” OR “magnetic resonance image” OR “brain imaging” OR “structural” OR “MRI” OR “functional MRI” OR “fMRI” OR “DTI” OR “diffusion tensor imaging” OR “quantitative MRI”) AND (“gray matter” OR “white matter” OR “ventricles “OR “central nervous system” OR “CSF” OR “Cerebral ventricles” OR “brain” OR “cortical thickness” OR “free water” OR “ventricular volume” OR “microstructure” OR “cognition”). We also followed the references of the relevant articles to find additional qualified studies. The data selection was based on PRISMA’s guidelines for preferred reporting of systematic reviews and meta-analyses (Liberati et al., 2009). After retrieving all the available studies and removing the duplicates, two independent authors screened the studies through their title and abstract. The potentially eligible studies then entered the process of checking the full texts by these authors. Any disagreement was solved by the third author.

**Fig. 1:**
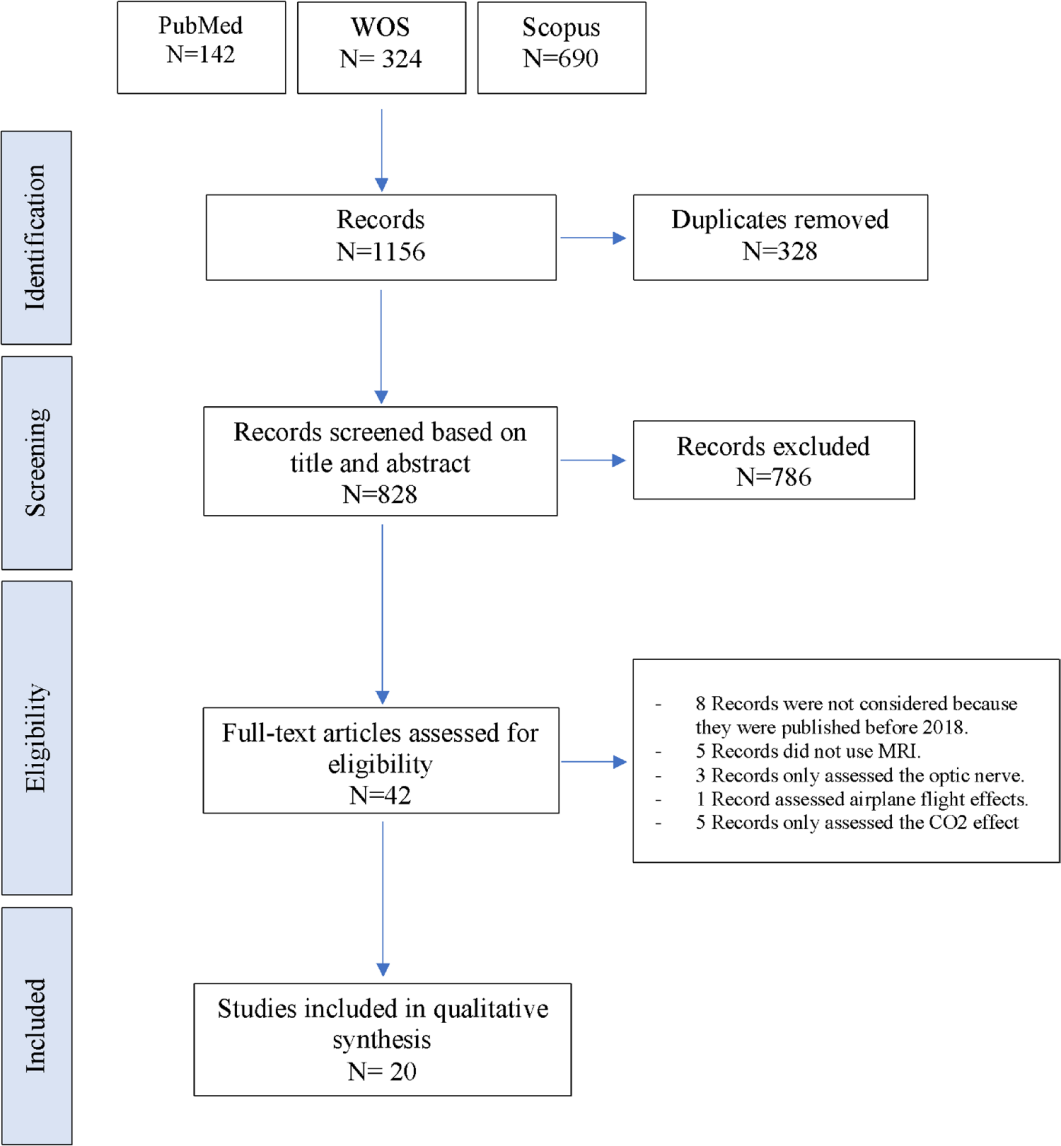
Flow diagram summarizing the selection of eligible studies based on the PRISMA guidelines

We included studies if: 1) they employed any MRI-based technique to assess the brain structure, microstructure, and function, after long duration or short duration spaceflight; 2) they were original peer-reviewed papers published in English. We excluded articles if 1) they employed imaging techniques other than MRI; 2) they did not directly assess MRI data; 3) they considered only alterations in optic nerve or just assessed the CO_2_ effect; 4) studies that didn’t involve humans; 5) the full-text article was not available. Our search was not limited to specific spaceflight experience or to a defined age range as we aimed at collecting all available evidence on the potential ability of MRI to assess brain changes in astronauts. The initial search resulted in 1156 papers, which were screened for eligibility, resulting in 42 records retrieved. A total of 20 met the inclusion criteria (Fig. 1). Details of the characteristics of the included studies are displayed in Table 1.

**Table 1:** Overview of Demographic and Neuroimaging data / findings of reviewed articles

Author/ year	Title	Number of participants (F/M) Age (mean ± SD)	Number of control group (F/M) Age (mean ± SD)	Imaging protocol	Anatomical region	Data collection interval	Findings
Hasan et al., 2018	Brain Quantitative MRI Metrics in Astronauts as a Unique Professional Group	N = 10 (0/10) 50.1 ± 2.5	–	Philips Intera 3.0 T-T1-weighted (3D-SPGR)-Turbo dual spin echo (DSE)-FLAIR-DTI QMRI	-White matter -Gray matter -CSF spaces	–	Some of the findings demonstrated statistically significant positive features suggestive of structural neuroplasticity, potentially associated with the professional activities of astronauts, and compensatory neurogenesis that counterweighs the expected normative volume loss with age. The observed relaxation time trend in the deep subcortical GM of astronauts was consistent with predicted non-heme iron content, implying normative cerebral iron metabolism in this group
Pechenkova et al., 2019	Alterations of Functional Brain Connectivity After Long-Duration Spaceflight as Revealed by fMRI	N = 11 (0/11) 45 ± 5	N = 11 (0/11) 44 ± 6	3 T GE-T2-weighted functional images-GRE EPI-T1-weighted (FSPGR) pre-central and post-central	-Cerebellum -Brain cortex	Pre-flight follow-up: 94 ± 36 days Post-flight follow-up: 9.4 ± 2.4 days Task of fMRI = activation	Changes in functional brain connectivity were observed in a group of cosmonauts after long-term spaceflight compared to a control group Increased connectivity was found between the left and right insulae as well as between the right posterior supramarginal gyrus within the TPJ region and the rest of the brain. A correlation was found between the severity of space motion sickness symptoms and the post-to-pre-flight difference in connectivity between the right posterior temporal cortex and the left anterior insula No alterations were found in activation elicited by the gait-like plantar stimulation.
Riascos et al., 2019	Longitudinal Analysis of Quantitative Brain MRI in Astronauts Following Microgravity Exposure	N = 19 (3/16) 50.6 ± SD not reported 10 astronauts with short-duration spaceflights and 9 astronauts with long-duration spaceflights	–	T2-weighted-FLAIR -3D T1w-MPRAGE-DWI-3 T	-Brain regions involved in visual function	Post-flight follow-up: 5 days	The study found that fractional anisotropy (FA) was reduced in the right posterior thalamic radiations, indicating white matter alterations in astronauts following spaceflight There was a trend of increase in the mean diffusivities of different subregions of the occipital cortex on the right side, including calcarine, middle occipital, inferior occipital, and fusiform gyri, although this trend became insignificant after the false discovery rate (FDR) correction Cortical thinning was observed in the right occipital lobe and bilateral fusiform gyri, suggesting potential structural changes in the visual domain cortices The left thalamus showed volume reduction, and there was a trend of increase in lateral ventricular volume in the postflight scans The study also noted a small increase in left lateral ventricular CSF volume in the postflight scans, potentially indicating changes in CSF reabsorption and intracranial CSF volume
Roberts et al., 2019	Prolonged Microgravity Affects Human Brain Structure and Function	N = 19 7 astronauts (1 woman; mean age: 46.7 ± 2.1) with short-duration spaceflights and 12 astronauts (2 women; mean age: 47.5 ± 4.8) long-duration spaceflights	–	T1-weighted 3 T	-White matter -Gray matter -Ventricles	–	No significant change was seen pre- to postflight in the total volume of gray matter or white matter for either the Shuttle or ISS astronauts In accordance with our prior study, there was a significant increase in the total ventricular volume postflight compared with preflight in the ISS astronauts but not in the Shuttle astronauts. The %DVV pre- to postflight was significantly associated with the flight duration across astronauts There was a significant negative association between astronauts’ ages and %DVV when adjusted for flight duration There was significant crowding of brain parenchyma at the vertex involving supplementary motor, premotor, and primary sensorimotor regions, consistent with our previously reported radiographic findings of central sulcus narrowing. There was also displacement of brain tissue along the ventricular margins, consistent with enlargement of the ventricular system No significant local changes in brain boundary were observed in the Shuttle astronauts following short-duration spaceflight. No significant association was seen between mission length and performance on any of the subtests of the WinSCAT. Brain structural changes are likely progressive based on mission duration.
Lee et al., 2019	Spaceflight-Associated Brain White Matter Microstructural Changes and Intracranial Fluid Redistribution	N = 7 (1/6) 47.2 + 1.5	N = 8 2/6 45.9 + 2.5	DWI-3 T	-White matter -CSF spaces	Pre-flight scans 274 days before launch (range: 44-627 days) and post-flight scans at a median of 6 days after return	There was a widespread increase in extracellular free water (FW) volume in the frontal, temporal, and occipital lobes, as well as a decrease in FW in the posterior aspect of the vertex. White matter changes were observed in the right superior and inferior longitudinal fasciculi, the corticospinal tract, and cerebellar peduncles. The magnitude of white matter changes was greater than those typically seen during the same period with healthy aging. Mission duration was associated with cerebellar white matter change, and white matter changes in the superior longitudinal fasciculus were associated with balance changes in astronauts.
Van Ombergen et al., 2019	Brain Ventricular Volume Changes Induced by Long-Duration Spaceflight	N = 11 (0/11) 44.6 ± 4.4	N = 11 (0/11) 43.6 ± 6.0	T1-weighted structural MRI (3 T)	-CSF spaces -Ventricles	Pre-flight scans 85 ± 34 days before launch, post-flight scans 9.9 ± 2.9 days after return, and follow-up scans 214 ± 45 days after return	Long-duration spaceflight induced significant changes in brain ventricular volume, specifically in the lateral ventricle, third ventricle, and total ventricular volume. There was no significant effect on the volume of the fourth ventricle. Post-flight measurements showed higher volumes in the lateral ventricle, third ventricle, and total ventricular volume compared to pre-flight values. Follow-up measurements revealed a decrease in the volume of the third ventricle and total ventricular volume compared to post-flight values. Comparison between pre-flight and follow-up measurements showed significant differences in the lateral ventricle, third ventricle, and total ventricular volume. The interaction effect of group and time was significant for the lateral ventricle, third ventricle, fourth ventricle, and total ventricular volume. There was no significant relationship between the percentage of total ventricular volume change and mission duration, age at launch, previous space experience, or total intracranial volume.
Kramer et al., 2020	Intracranial Effects of Microgravity: A Prospective Longitudinal MRI Study	N = 11 (1/10) 45 ± 5	–	T1-weighted 3 T	-White matter -Ventricles -CSF spaces	Post-flight follow-up scans 1, 30, 90, 180, and 360 days after landing Pre-flight scans 530 ± 190 days prior to launch	The paper concludes that continued research into the pathophysiologic causes of ventricular enlargement in astronauts.
Jillings et al., 2020	Macro- and Microstructural Changes in Cosmonauts’ Brains after Long-duration Spaceflight	N = 11 (0/11)	–	DWI-3 T T1-weighted	-White matter -Gray Matter CSF spaces	Pre-flight scans 9 days before launch, post-flight scans 9 days after return, and follow-up scans 7 months after return	Increased WM in the cerebellum after spaceflight, providing the first clear evidence of sensorimotor neuroplasticity. A widespread redistribution of CSF, with concomitant changes in the voxel fractions of adjacent GM GM changes are the result of morphological changes rather than net tissue loss, which remained unclear from previous studies. Evidence of spaceflight-induced neuroplasticity to adapt motor strategies in space and evidence of fluid shift-induced mechanical changes in the brain. Whole-brain analysis of brain tissue changes after spaceflight Spaceflight induces reversible changes in CSF, GM, and WM VFs Spaceflight induces local brain volume changes Brain tissue changes after spaceflight are not attributed to aging
Hasan et al., 2018	The Impact of 6 and 12 Months in Space on Human Brain Structure and Intracranial Fluid Shifts	N(6-month) = 10 (1/9) 48 ± 6 N(12-month) = 10 (9/1)	N(T1-weighted) = 20 (10/10) 59 ± 6.7 N(dMRI) = 14 (10/4) 67 ± 2.8	T1-weighted structural MRI Diffusion-weighted 3 T	-Ventricles -Cerebellum -Cortex -Gray matter	Pre-flight scans 60 days before launch and post-flight scans 5, 30, 90, and 180 days after return	12-month missions in space resulted in larger changes in brain structure and fluid shifts compared to 6-month missions and aging. Changes in brain areas and fluid shifts generally returned to pre-flight levels by 6 months after flight. Ventricular volume substantially increased for both 12-month and 6-month astronauts, with little recovery at 6 months post-flight. The slopes of change in brain measures for the 6-month astronauts were significantly greater than those of control subjects, indicating more pronounced changes. The 12-month astronauts exhibited even steeper slopes of change compared to controls. The 6-month astronauts showed a significant return toward pre-flight levels in the 6 months post-flight for certain brain regions. The 12-month astronauts also exhibited pre-to-post-flight changes followed by recovery at 6 months post-flight.
Marshall-Goebel et al., 2021	Association of Structural Changes in Brain and Retina after Long Duration Space Flight	N = 19 (5/14) 45.2 ± 6.4	–	T1-weighted structural MRI 3 T	-Retinal thickness -Ventricles -CSF Spaces	Pre-flight scans 324 ± 229 days before launch and post-flight scans 3 ± 1 days after return	Of the 19 crew members included in this analysis, 5 (26.3%) were women and 14 (73.7%) were men. The mean (SD) age was 45.2 (6.4) years, height was 176.1 (6.5) cm, and weight was 76.1 (9.4) kg. Forty-one healthy non–crew members were included in the analysis for comparison of lateral ventricle volumes (mean [SD] age, 46 [5.8] years; age range, 36-57 years). When adjusted for preflight TRT, the model predicted that for each 1-mL increase in lateral ventricle volume after spaceflight, the post flight TRT increased 4.7 μm (95% CI, −1.5 to 10.8 μm; P = .13); however, there was no association between post flight TRT and change in white matter volume (0.02 μm; 95% CI, −0.5 to 0.5 μm; P = .94) or intracranial volume change (ie, total brain tissue plus CSF; 0.02 μm; 95% CI, −0.6 to 0.6 μm; P = .95) Adjusting for spaceflight mission duration improved the prediction of post flight TRT to a mean of 5.1 μm (95% CI, −0.4 to 10.5 μm; P = .07) per 1-mL of lateral ventricle volume increase, although still not reaching traditional levels of statistical significance. One participant was diagnosed as having bilateral Frisen grade 1 ODE after the flight. Absolute ventricular volume after spaceflight remained within normal population sizes and did not approach the magnitude of that observed in terrestrial pathologies
Roberts et al., 2021	Longitudinal Change in Ventricular Volume is Accelerated in Astronauts Undergoing Long-Duration Spaceflight	N = 18 (3/15) 48.43 ± 4.35	N (healthy age- and sex-matched adults) = 18 (3/15) 51.26 ± 3.88 N (older adults) = 79 (13/66) 73.26 ± 5.34	3 T, 1.5 T	-Ventricles	Pre-flight scan before launch and post-flight scan withing the two weeks after return	The astronauts had an annual rate of ventricular expansion more than three times that expected from normal aging Ventricular enlargement post-flight may be a feature of Spaceflight Associated Neuro-ocular Syndrome
Koppelmans et al., 2022	Cortical Thickness of Primary Motor and Vestibular Brain Regions Predicts Recovery from Fall and Balance Directly after Spaceflight	N = 14 (2/12) 52.5 ± 5.4	–	T1-weighted structural MRI T2-weighted Diffusion-weighted 3 T	-Gray matter -White matter -Cortex -Cerebellum	Pre-flight scans 90 days before launch and post-flight scans 60 days after return	Motor adaptations to the microgravity environment during spaceflight allow astronauts to perform adequately in this unique environment. Upon return to Earth, this adaptation is no longer appropriate and can be disruptive for mission critical tasks. Structural and diffusion MRI scans from 14 astronauts collected before launch, and motor measures (balance performance, speed of recovery from fall, and tandem walk step accuracy) collected pre-flight and post-flight were analyzed. Regional measures of gray matter volume (motor cortex, paracentral lobule, cerebellum), myelin density (motor cortex, paracentral lobule, corticospinal tract), and white matter microstructure (corticospinal tract) were derived as a-priori predictors. Whole-brain analyses showed that paracentral and precentral gyri thickness significantly predicted recovery from fall post-spaceflight. Thickness of vestibular and sensorimotor regions, including the posterior insula and the superior temporal gyrus, predicted balance performance post-flight and pre-to-post-flight decrements. Greater cortical thickness pre-flight predicted better performance post-flight. Regional thickness of somatosensory, motor, and vestibular brain regions has some predictive value for post-flight motor performance in astronauts, which may be used for the identification of training and countermeasure strategies targeted for maintaining operational task performance
Hupfeld et al., 2022b	Brain and Behavioral Evidence for Reweighting of Vestibular Inputs with Long-Duration Spaceflight	N = 15 (0/15) 47.5 ± 6.3	N = 14 (0/14) 42.0 ± 9.7	T1-weighted structural MRI T2-weighted fMRI 3 T	-Task based fMRI -Vestibular simulation	Pre-flight scans 180 and 60 days before launch and post-flight scans 4, 30, 90, and 180 days after return	The study found that spaceflight led to reductions in somatosensory and visual cortical deactivation, indicating sensory compensation and reweighting with microgravity. These changes in brain activity correlated with changes in eyes closed standing balance, suggesting a relationship between neural processing and behavioral performance No significant longitudinal changes in cerebellar activity during vestibular stimulation were observed. However, at a more liberal statistical threshold, a relationship emerged between flight duration and pre-to-post flight deactivation in regions of the cerebellum. Those who completed shorter flights exhibited decreased deactivation, while those who completed longer flights showed increased deactivation. These results should be interpreted with caution and further examined in future studies,
Doroshin et al., 2022	Brain Connectometry Changes in Space Travelers After Long-Duration Spaceflight	N = 12 (0/12) 45 ± 5	N=N/A 43 ± 6	Diffusion-weighted 3 T	-White matter	Pre-flight scans 89 days before launch, post-flight scans 10 days after return, and follow-up scans 230 days after return	The study found significant microstructural changes in several large white matter tracts, such as the corpus callosum, arcuate fasciculus, corticospinal, corticostriatal, and cerebellar tracts, in cosmonauts after long-duration spaceflight. The connectometry analysis showed significant results for increasing quantitative anisotropy (QA) in the forceps minor, which mediates the connectivity of the inferior-lateral and orbital parts of the brain. The study used diffusion magnetic resonance imaging (dMRI) data and differential tractography to investigate specific tracts that exhibit structural changes after spaceflight. The quantitative anisotropy (QA) was used as the local connectome fingerprint (LCF) in the connectometry analysis, which correlated QA changes with days in space for each cosmonaut. The false discovery rate (FDR) analysis was used to determine the significance of findings, with FDR 0.05 considered highly confirmatory and FDR 0.3 showing non-significant findings. Overall, the study identified significant microstructural changes in white matter tracts and demonstrated the potential of connectometry analysis to investigate brain changes after spaceflight.
Van Ombergen et al., 2018	Brain Tissue–Volume Changes in Cosmonaut	N = 10 (0/10) 44	–	T1-weighted MRI	-Gray matter -White matter -CSF spaces	Pre-flight scans before launch, post-flight scans 9 days after return, and follow-up scans 209 days after return	Spaceflight leads to an upward shift of the cerebral hemispheres, a decrease in frontotemporal volume, and an increase in ventricle size. Most of the loss in gray matter volume seen immediately post flight had recovered to preflight levels, while CSF volume continued to increase in the subarachnoid compartment. CSF spaces expand even many months after a return to Earth, suggesting a persistent disturbance in CSF circulation. These brain-volume changes may relate to ocular and visual abnormalities after long-duration spaceflight. Topologic patterns of volume change in gray matter and CSF spaces were observed in cosmonauts, with decreased gray-matter volume in certain regions post flight and persistent reduction in ventral cortical regions at long-term follow-up.
Hupfeld et al., 2022a	Longitudinal MRI-Visible Perivascular Space (PVS) Changes with Long-Duration Spaceflight	N = 15 (4/11) 47.46 ± 6.28	N = 11 (3/8) 42.28 ± 10.60	T1-weighted structural MRI (3.0 T)	-Gray matter -White matter -CSF spaces	Pre-flight scans 180 and 60 days before launch, post-flight and follow-up scans 4, 30, 90, and 180 days after return	The study included 15 astronauts, with 9 being novice astronauts and 6 being experienced astronauts. The experienced astronauts were older on average. No other significant differences were found between the novice and experienced groups in terms of sex, mission duration, or time between landing and the first post-flight MRI scan. The automated segmentation algorithm used to calculate perivascular space (PVS) metrics performed similarly to visual ratings, validating its accuracy. Four out of five PVS metrics showed high reliability across pre-flight and control time points. Novice astronauts showed an increase in total PVS volume from pre-to post-flight, while experienced astronauts did not. This suggests that experienced astronauts may exhibit holdover effects from prior spaceflights. There was a positive correlation between pre-flight PVS load and prior flight experience, although it did not reach statistical significance. Changes in ventricular volume were not significantly associated with changes in PVS characteristics, and the presence of spaceflight-associated neuro-ocular syndrome (SANS) was not associated with PVS number or morphology.
Jillings et al., 2023	Prolonged Microgravity Induces Reversible and Persistent Changes on Human Cerebral Connectivity	N = 13 (0/13) 45.0 ± 2.4	N = 14 0/14 41 ± 4	Resting-state fMRI T1-weighted structural MRI T2-weighted 3 T	-Gray matter -White matter -CSF spaces	Pre-flight scans 96 ± 26 days before launch, post-flight scans 9 ± 1 days after return, and follow-up scans 239 ± 45 days after return	Prolonged microgravity induces reversible and persistent changes in human cerebral connectivity. fMRI was used to assess global connectivity changes over time in cosmonauts before, shortly after, and eight months after spaceflight. Persisting connectivity decreases were observed in the posterior cingulate cortex and thalamus, while persisting increases were observed in the right angular gyrus. Connectivity in the bilateral insular cortex decreased after spaceflight but reversed at follow-up. No significant connectivity changes were found in a matched control group. Exposure to prolonged microgravity influenced longitudinal functional connectivity in a heterogeneous manner, decreasing overall connectivity in some areas and increasing it in others. There was extreme evidence for a change in time in cosmonauts, indicating that the effects observed in the cosmonaut cohort can be attributed to prolonged microgravity. The pre-to-post flight change in cosmonauts differed from the effect over time in controls.
Burles et al., 2023	The Unresolved Methodological Challenge of Detecting Neuroplastic Changes in Astronauts	N = 43 (10/33) 47.79 ± 5.06	–	T1-weighted structural MRI T2-weighted FLAIR 3 T	-Gray matter	Pre-flight scans 381.69 ± 213.66 days before launch, and post-flight scans 6.68 ± 5.79 days after return	Astronauts display an upward shift in the position of the brain within the skull and a redistribution of cerebrospinal fluid after spaceflight. Magnetic resonance imaging studies have reported local changes in brain volume, which have been interpreted as a neuroplastic response to spaceflight. However, it is suggested that the grey matter volume changes detected using standard processing pipelines for neuroimaging analyses could be contaminated by errors in tissue segmentation, undermining the interpretation of these changes as direct evidence of neuroplastic adaptation. Voxel-based morphometry analysis following unimodal segmentation detected widespread volumetric changes associated with spaceflight, including grey matter losses in multiple locations across the occipital, temporal, and frontal lobes. However, the multimodal segmentation paradigm failed to detect these grey matter losses and only identified grey matter gains in specific regions. The unimodal analysis detected more extensive grey matter gains compared to the multimodal analysis. Errors in tissue segmentation can lead to mischaracterization of dural structures as grey matter, resulting in misclassification of tissue types. Utilizing alternate structural imaging modalities, selecting the highest-performing segmentation approaches, and developing novel approaches that perform best with a given imaging modality may help resolve artifacts associated with partial voluming errors.
Salazar et al., 2023	Changes in Working Memory Brain Activity and Task-Based Connectivity after Long-Duration Spaceflight	N = 15 (11/4) 47.5 ± 6.3	–	Task based fMRI (SWM task) T2-weighted 3 T	-Whole brain -Cerebellum	Pre-flight scans 180 and 60 days before launch, post-flight and follow-up scans 4, 30, 90, and 180 days after return	The study found no significant effects of spaceflight on spatial working memory (SWM) performance. However, there were significant changes in brain connectivity after spaceflight. The superior occipital gyrus showed reduced task-based connectivity with the rest of the brain, and there was decreased connectivity between the left middle occipital gyrus and other brain regions during SWM performance. Increased visual and visuomotor connectivity were correlated with improved SWM performance after spaceflight, while decreased visual and visual-frontal cortical connectivity were associated with poorer performance. These results suggest that while SWM performance remains consistent, there are underlying changes in connectivity among supporting networks, indicating both disruptive and compensatory alterations due to spaceflight.
McGregor et al., 2023	Impacts of Space Flight Experience on Human Brain Structure	N = 30 22 long-duration (6/16) 46.8 ± 5.6 8 short-duration 1/7 48 ± 2.3	–	T1W DWI 3 T	-Gray matter -White matter -Ventricles	Pre-flight scans before launch, post-flight scans 12 ± 6.3 days after launch for short-duration astronauts and 4.1 ± 1.8 days after launch for long-duration astronauts	Spaceflight induces gray matter shifts, free water redistribution, and ventricular enlargement. Longer missions induce greater fluid shifts. No differences in structural brain changes between novice and experienced astronauts. Greater prior fight history associated with decreases in FW (free water) following spaceflight Greater ventricular expansion for astronauts with longer inter mission intervals.

Abbreviations: *CI* confidence interval, *CSF* cerebrospinal fluid, *DTI* Diffusion tensor imaging, *DVV* percentage total ventricular volume change, *DWI* diffusion-weighted imaging, *fMRI* Functional magnetic resonance imaging, *FLAIR* Fluid-attenuated inversion recovery, *F/M* female-to-male ratio, *GM* gray matter, *ISS* international space station, *MRI* magnetic resonance imaging, *ODE* optic disc edema, *QMRI* quantitative magnetic resonance imaging, *SD* standard deviation, *TIT* total retinal thickness, *TPJ* temporoparietal junction, *WM* white matter

## Results

###  Characteristics of the studies

**Fig. 2:**
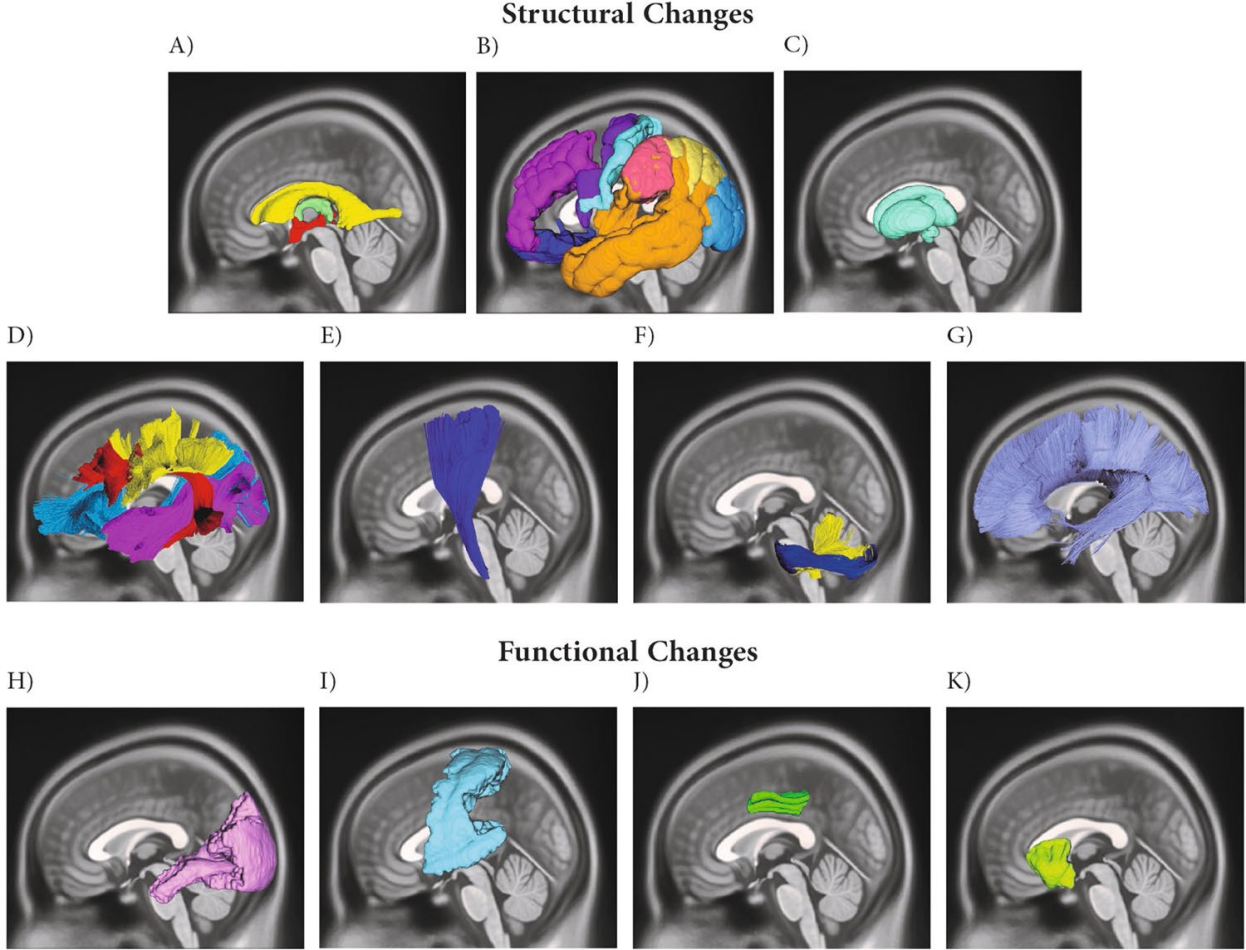
Summary of the structural and functional changes in brain after spaceflight. The structural changes included alterations in **A**) Ventricles and cerebrospinal fluid spaces, **B**) Cortical gray matter, including frontal, temporal, occipital, and parietal cortices, **C**) Subcortical gray matter, including thalamus **D**) Association fibers, including superior longitudinal fasciculus, inferior longitudinal fasciculus, and inferior fronto-occipital fasciculus, **E**) Corticospinal tract, **F**) Cerebellar peduncles, and **G**) Corpus callosum. The functional changes included alterations in H) Visual pathways, I) Motor pathways, **J**) Posterior cingulate cortex, and K) Insula. The picture was illustrated using DSI Studio (https://dsi-studio.labsolver.org/)

Our search returned a total of 1156 journal articles, of which twenty studies fulfilled the eligibility criteria and entered full-text data extraction. The field strength of either 3.0 or 1.5 Tesla was applied for MRI. Eight investigations used conventional T1/T2 weighted sequences of structural MRI to assess brain volume, density, and/or cortical thickness in astronauts (Van Ombergen et al., 2019; Van Ombergen et al., 2018; Hupfeld et al., 2022a; Burles et al., 2023; Roberts et al., 2019; Roberts et al., 2021; Kramer et al., 2020; Marshall-Goebel et al., 2021), two used diffusion-based MRI features (Doroshin et al., 2022; Lee et al., 2019), one research applied fMRI (Salazar et al., 2023), six used both structural MRI and diffusion-based MRI features (Riascos et al., 2019; McGregor et al., 2023; Hupfeld et al., 2020; Hasan et al., 2018; Koppelmans et al., 2022; Jillings et al., 2020), and three investigations employed both structural MRI and fMRI (Jillings et al., 2023; Pechenkova et al., 2019; Hupfeld et al., 2022b). Finally, among the included studies, nine studies recruited controls to find the effects of spaceflights on the brain of astronauts (Van Ombergen et al., 2019; Hupfeld et al., 2022a; Roberts et al., 2021; Doroshin et al., 2022; Lee et al., 2019; Hupfeld et al., 2020; Jillings et al., 2023; Pechenkova et al., 2019; Hupfeld et al., 2022b), while the rest merely compared pre-spaceflight and post-spaceflight profiles. We demonstrated a summary of the findings of the included studies (Fig. 2).

### Structural MRI

#### Studies by Ombergen et al.

**Fig. 3:**
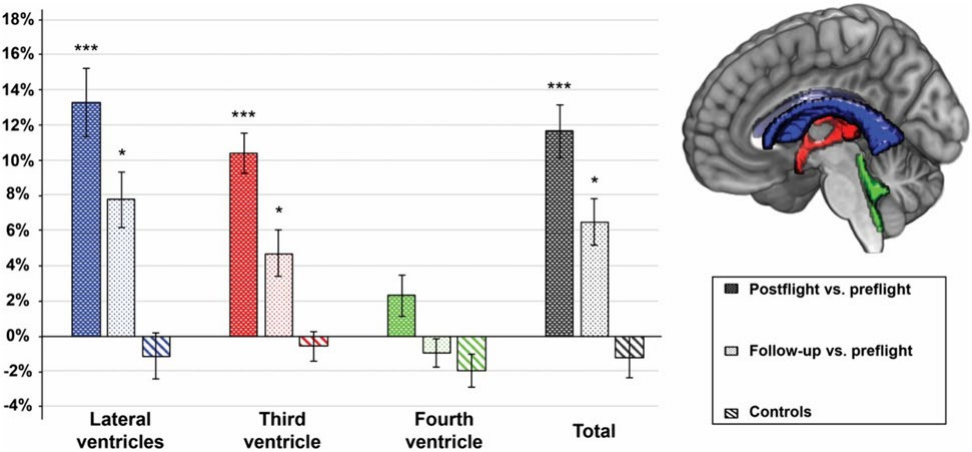
For comparisons postflight vs. preflight (dark-colored bar) and follow-up vs. preflight (light-colored bar), volume changes of each ventricular CSF compartment are shown. The figure was adapted from Van Ombergen et al. article published by PNAS with permission

In their studies, utilizing T1-weighted MRI scans to investigate brain structures before, immediately after, and seven months following long-duration spaceflight, Van Ombergen et al., 2019 observed significant expansions in the lateral and third ventricles right after the spaceflight. Additionally, they noted substantial increases in ventricular volumes. By the time of the seven-month follow-up, there was a discernible trend toward normalization of the initial postflight changes, though enlarged ventricular volumes remained (Fig. 3). In related research by the same group (Van Ombergen et al., 2018), they found that immediately after spaceflight, there was a reduction in gray matter volume in the orbitofrontal and temporopolar regions. In parallel, the volumes of the cerebral ventricles and basal cisterns saw an increase. By the long-term follow-up (average: 209 days post-spaceflight), the majority of the gray matter volume reductions had returned to their preflight levels. However, persistent reductions were observed in the ventral cortical region. It’s noteworthy that even though the ventricle CSF volume had gone back to preflight values, the subarachnoid CSF space around the brain continued to show an enlarged state.

#### Studies by Roberts et al.

Roberts et al. 2019 investigated changes in astronaut brain structures from pre- to postflight. In their whole-brain assessment, they performed volumetric analysis, local crowding or displacement of brain tissue along the brain-CSF interface, and regional deformation of the brain parenchyma. Their findings indicated that long-duration spaceflights (average: 162 days) led to an increase in the total ventricular volume, a change not observed in astronauts from short-duration Space Shuttle flights. Alongside these volumetric changes, they noticed structural alterations in the brain. This included the crowding of brain tissue at the topmost part (vertex) and modifications in white matter regions. These anatomical changes were correlated with poorer postural control, longer completion time of complex motor tasks, and faster reaction time in cognitive tasks (code substitution and continuous performance). Another study showed that the rate of change in ventricular volume over time for NASA astronauts was considerably more significant than that observed in healthy adults matched for age and sex, as well as in healthy older adults (Roberts et al., 2021). Interestingly, at the outset (baseline MRI), there was no notable difference in the total ventricular volumes between the control group (healthy adults) and astronauts who had been on the ISS. However, when compared to both the ISS astronauts and the control group, the older adults exhibited considerably larger total ventricular volumes.

#### Studies by Burles et al. and Kramer et al.

Following spaceflight, notable changes have been observed in the positioning and fluid dynamics of astronauts’ brains. One such observation is the upward shift of the brain within the cranial cavity, coupled with a redistribution of cerebrospinal fluid (Burles et al., 2023). Using voxel-based morphometry analysis after unimodal segmentation, there were discernible gray matter losses in the occipital, temporal, and frontal brain regions linked to the effects of spaceflight. Kramer et al., 2020, in a prospective longitudinal MRI study, highlighted a relationship between long-duration spaceflight and specific brain changes. These changes encompass increased pituitary gland deformation, amplified hydrodynamics of the aqueductal CSF, and a rise in the total volumes of both brain and CSF. Notably, on the first day after returning from their mission, astronauts exhibited increased average volumes in various brain compartments, including the overall brain, white matter, and lateral ventricles. These volume increases, including the combined brain and CSF volumes, persisted for durations extending up to a year.

#### Studies by Hupfeld et al. and Marshall-Goebel et al.

Interestingly, first-time astronauts showed an increased perivascular space (PVS) volume after their mission compared to before their flight. In contrast, astronauts with prior spaceflight experience did not exhibit this change, suggesting possible residual effects from their previous missions. While there appeared to be a connection between a higher PVS volume and prior flight experience, this correlation did not achieve statistical significance. Additionally, the alterations in ventricular volume from pre- to post-flight did not correspond significantly with changes observed in PVS characteristics (Hupfeld et al., 2022a). Utilizing both optical coherence tomography (OCT) and MRI, (Marshall-Goebel et al. 2021) identified a potential correlation between alterations in retinal thickness and the volume of the lateral ventricles due to spaceflight. However, this relationship was not conclusively established. It’s also important to note that the changes in total retinal thickness induced by spaceflight did not correlate with variations in white matter volume or the combined volume of brain tissue and cerebrospinal fluid.

### Diffusion-weighted MRI

#### The study by Lee et al.

Using DWI, significant associations were found between mission duration and changes in fractional anisotropy (FA) as well as between preflight to postflight alterations in extracellular free water (FW) and the total number of missions undertaken (Lee et al., 2019). Lee et al. identified an increase in FW volume within the frontal, temporal, and occipital lobes. Conversely, there was a decrease in FW towards the posterior aspect of the vertex. Post-spaceflight scans also revealed a decrease in FA in various regions, including the right superior longitudinal fasciculus, inferior longitudinal fasciculus, inferior fronto-occipital fasciculus, corticospinal tract, and both the inferior and middle cerebellar peduncles. Conversely, there was an increase in radial diffusivity (RD) in white matter regions beneath the precentral, postcentral, supramarginal, and angular gyrus. Furthermore, certain areas, particularly the superior longitudinal fasciculus (SLF), exhibited reduced axial diffusivity (AD) values.

#### The study by Doroshin et al.

Doroshin et al. 2022 employed diffusion-based MRI analysis combined with differential tractography to assess the effects of spaceflight on the white matter of astronauts. Their findings revealed significant increases in quantitative anisotropy (QA) within various white matter tracts post-long-duration spaceflight. Tracts affected included the arcuate fasciculus, cerebellum, corpus callosum, corticospinal tract, and corticostriatal tract. Intriguingly, there was an effect of time on specific QA changes; the corticostriatal tracts and frontal crossing fibers (forceps minor and corpus callosum body) showed QA reductions from pre- to post-spaceflight and elevations from post-flight to follow-up (average: 230 days pos-spaceflight). Furthermore, their connectometry analysis discerned post-flight QA increases specifically in the corpus callosum, corticopontine tract, and superior longitudinal fasciculus.

### Functional MRI, the study by Salazar et al.

Salazar et al. 2023, delved into the effects of spaceflight on brain connectivity by utilizing task-based fMRI analyses. Notable alterations were observed in the superior occipital gyrus when comparing the pre- and post-spaceflight images. It exhibited reduced task-based connectivity with other parts of the brain, which suggests a heightened modularity within the visual network. Additionally, other regions, including the left middle occipital gyrus, the left parahippocampal gyrus, the left cerebellum, and the left lateral occipital cortex, similarly displayed diminished connectivity, especially during spatial working memory (SWM) tasks. Interestingly, the study also found that improved SWM performance was associated with an increase in visual and visuomotor connectivity. On the other hand, degraded performance in SWM was linked with a decrease in connectivity between the visual and visual-frontal cortical regions. While the overall performance in SWM remained consistent among the astronauts, the changes in connectivity within the networks that support this function indicated a combination of both disruptive and compensatory neural alterations post-flight. In their study, they included mean-centered age, sex, flight duration, and the time between landing and the test date as covariates to assess the direct effects of the spaceflight. This suggests that while spaceflight may introduce changes to brain connectivity patterns, the brain may employ compensatory mechanisms to maintain cognitive task performance.

### Structural MRI and diffusion-weighted MRI

#### The study by Hupfeld et al.

Hupfeld et al. 2020 made an intriguing observation regarding the astronauts who were in space for both 6 and 12 months. Both groups experienced significant increases in their ventricular volume. What’s more fascinating is that there seemed to be minimal recovery even 6 months after their return. When investigating the supplementary motor area’s gray matter volume, a pattern emerged: the 6-month astronauts displayed steeper slopes of change when compared to the control group. However, those astronauts who were in space for 12 months had even more dramatic changes; they showed steeper slope of the pre- to postflight changes in the right supplementary motor area gray matter volume alongside right precentral gyrus gray matter volume and cortical thickness.

#### The study by McGregor et al.

On a similar note, (McGregor et al., 2023) embarked on a comprehensive study that aimed at merging diffusion-based MRI data with structural data derived from T1-weighted sequences of MRI. Their findings corroborate what Hupfeld et al. discovered concerning ventricular expansion: the right lateral and third ventricles of the brain tended to expand more with lengthier space missions. Remarkably, the bulk of this expansion happened during the initial 6 months in space. Furthermore, McGregor et al. found that spaceflight seemed to prompt an increase in the gray matter volume at the apex of the brain in contrast to its base. Another critical finding highlighted the extracellular free water fractional volume. During the astronauts’ stint in space, there was a noticeable decrease in this volume in the vertex of the brain and an increase in the temporal and frontal lobes. These findings give a more detailed insight into the brain’s adaptive responses to the conditions of spaceflight, emphasizing both the resilience and vulnerability of various brain regions and microstructures.

#### The study by Jillings et al.

One of the profound discoveries by (Jillings et al., 2020) was the enhancement of white matter within the cerebellum. Even after returning to the gravitational constraints of Earth, this modification persisted for a remarkable 7 months, hinting at lasting changes in the astronauts’ neural architecture. Moreover, there were notable shifts in the distribution of the CSF, which also influenced neighboring gray matter voxels. This highlights the intricate interplay between various components of the central nervous system and how they might reconfigure themselves in response to the altered gravitational forces of space.

#### The study by Koppelmans et al.

**Fig. 4:**
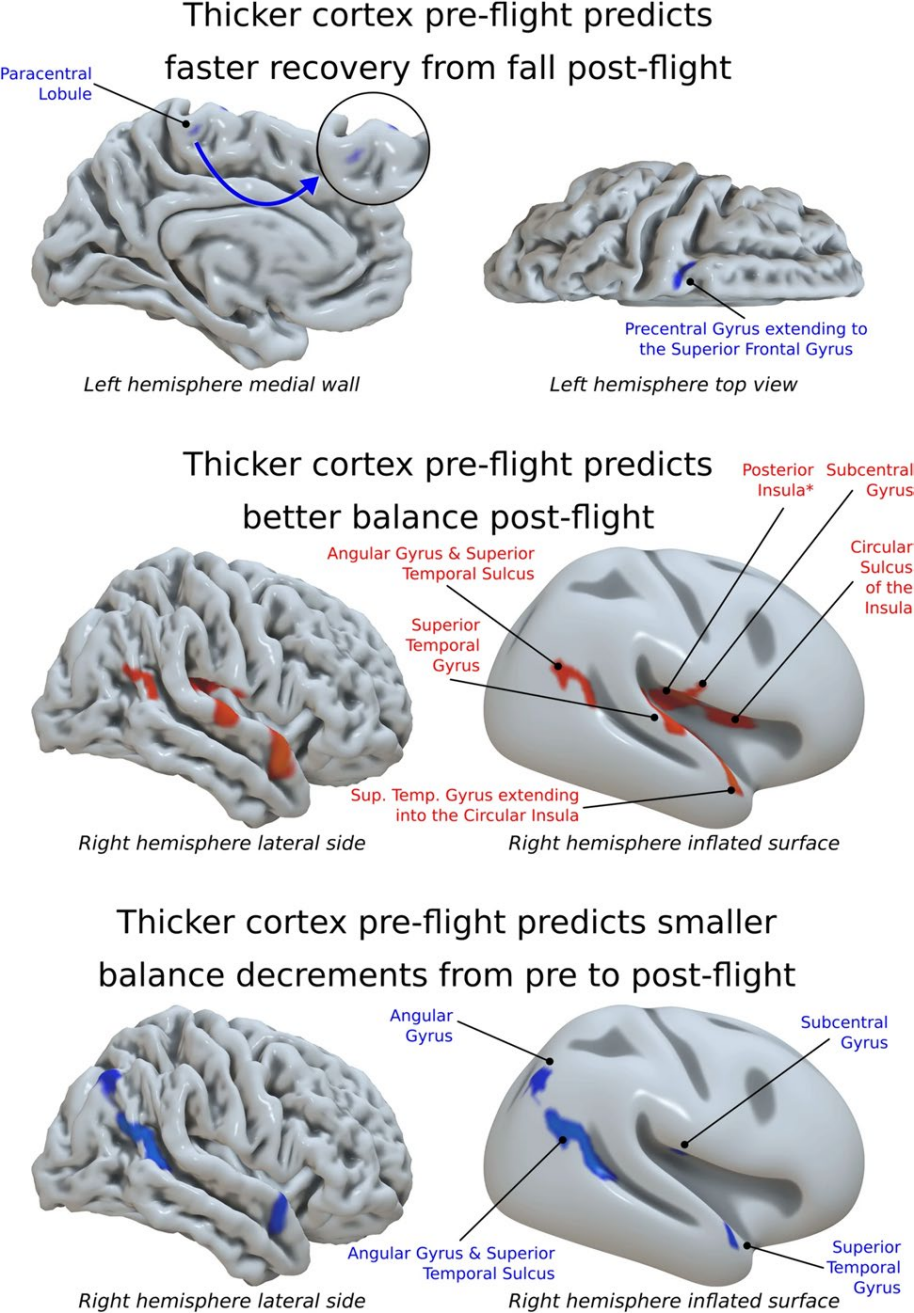
Correlation between pre- and post-flight measurements of Cortical Thickness (rows 1 and 2), or b, motor performance changes from pre- to post-flight (row 3). The figure was adapted from Koppelmans et al. article published by Springer Nature with permission

When delving deeper into how these structural brain changes impact post-flight motor performance, (Koppelmans et al., 2022) performed a meticulous examination. Surprisingly, despite comprehensive regional analyses of gray matter volume, myelin density, and white matter microstructure, no direct correlations with motor performance were identified. However, the thickness of the paracentral and precentral gyri, areas that play pivotal roles in motor processing, notably predicted recovery from falls post-spaceflight (Fig. 4). Moreover, the posterior insula and the superior temporal gyrus, emerged as predictors of post-flight balance challenges. Furthermore, the study also indicated that spaceflight expanded the volume of the paracentral lobule and led to a decrease in the integrity of white matter microstructures in vital pathways like the corticospinal tract and cerebellar peduncles. These alterations might have implications for the post-fall balance improvement in astronauts after the spaceflight.

#### The study by Hasan et al.

Hasan et al.’s (2018) study is a testament to this advanced multi-modal approach. Their work hints at an astonishing capability of the astronaut’s brain: structural neuroplasticity. Their study suggests that the brain is molding itself in ways potentially linked to the astronauts’ unique professional tasks in space. Equally striking is the discovery related to compensatory neurogenesis. As people age, it’s normal for the brain to lose volume (Fujita et al., 2023). Yet, Hasan et al. found that astronauts seem to be pushing back against this natural decline. Their brains exhibit signs of neurogenesis or new neuron formation, which might counteract the expected age-related volume loss. Additionally, the deep subcortical gray matter in astronauts presented normative cerebral iron metabolism, which is consistent with the presence of non-heme iron. Imaging iron using T2, they found a trend in relaxation times (hippocampus > amygdala > caudate nucleus > putamen > globus pallidus), as expected.

#### The study by Riascos et al.

Riascos et al. (2019), on the other hand, turned their focus to regions of the brain vital for vision. Using DTI, the team mapped out alterations in the brain’s white matter tracts. Post-spaceflight, there were specific changes in areas involved in visual function. This was evident from the decreased FA values, especially in the right posterior thalamic radiations. Simultaneously, there was an increase in mean diffusivity (MD) in specific parts of the occipital cortex. Moreover, volumetric analysis brought more changes to light, such as cortical thinning in the visual processing areas and an increase in lateral ventricular volume. The authors found that whether astronauts went on short or long voyages to space, the astronauts exhibited comparable alterations in brain microstructure. Taken together, these advanced MRI-based investigations paint a picture of the brain’s remarkable adaptability and coping with the unique challenges of space.

### Structural MRI and functional MRI

#### The study by Pechenkova et al.

**Fig. 5:**
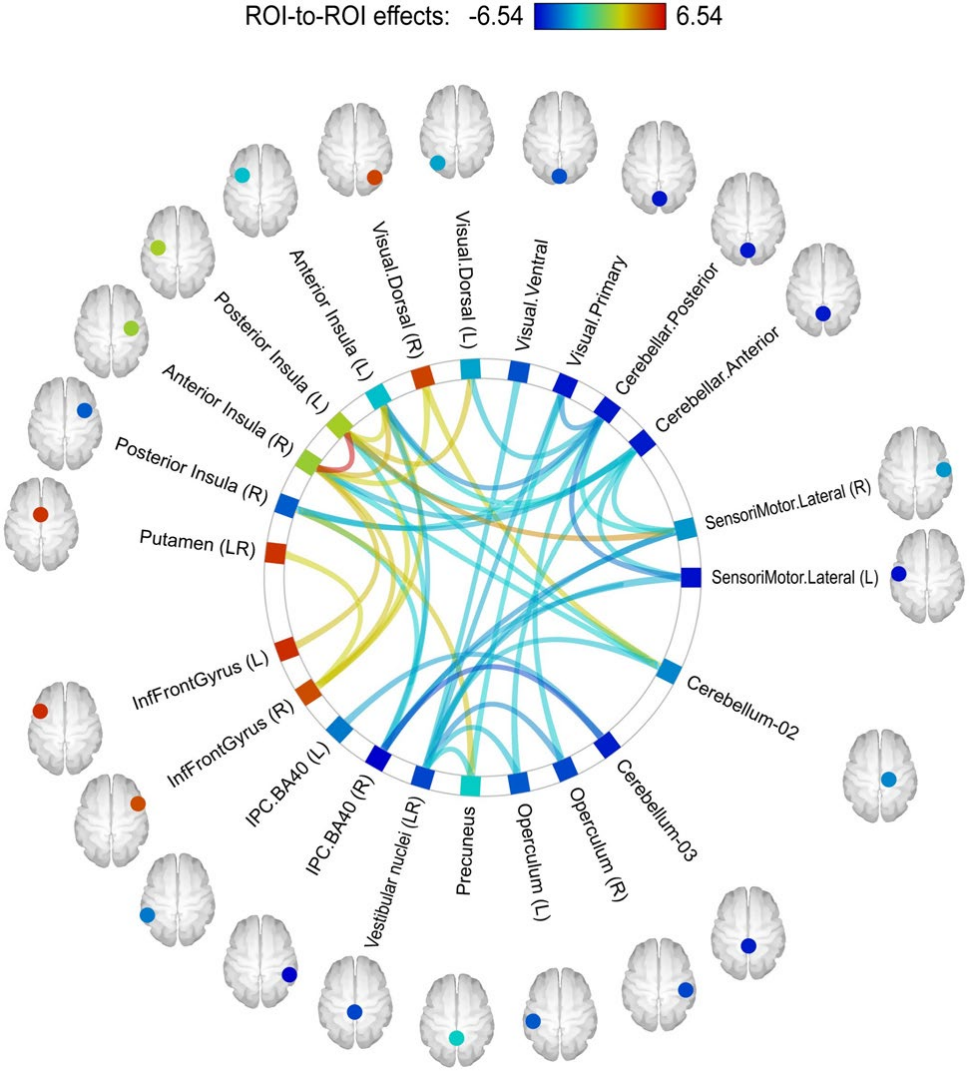
In the post-flight comparison of cosmonauts versus controls, the ROI-to-ROI connectivity analysis of the NBS network identified a subnetwork demonstrating changes in PPI connectivity values. The figure was adapted from Pechenkova et al. article published by Frontiers with permission

Some recent studies underscore how space travel can induce profound changes in the functional connectivity of the brain, and how these alterations might relate to observable behaviors. In Pechenkova et al.’s study (Pechenkova et al., 2019), the use of a plantar stimulation task elucidated the difference in functional connectivity between astronauts after long-term spaceflight and a control group (Fig. 5). The study’s key findings were the disconnections between several regions involved in motor, somatosensory, visual, and vestibular processing. This points towards a significant rearrangement in the functional connectivity within these vital networks. Interestingly, the increase in connectivity between the right and left insulae and between parts of the right posterior supramarginal gyrus and the rest of the brain may suggest compensatory mechanisms.

#### The study by Hupfeld et al.

Hupfeld et al.’s investigation(Hupfeld et al., 2022b) offers insights into the repercussions of altered vestibular signaling in space. The noted decrease in behaviors mediated by the vestibular system, such as maintaining balance, signifies the challenges astronauts face when readapting to Earth’s gravitational environment. It’s notable that there was no significant change in cerebellar activity, a primary site for processing vestibular inputs, during vestibular stimulation. This finding suggests that other areas of the brain might undergo more pronounced changes to account for the altered vestibular signaling in space.

#### The study by Jillings et al.

Jillings et al.’s study (Jillings et al., 2023) further elucidates the complexities of spaceflight’s impact on brain connectivity. Their findings indicate that the effects of prolonged microgravity on the brain are quite diverse. Some brain areas like the posterior cingulate cortex and thalamus witnessed persistent connectivity decreases. At the same time, areas like the right angular gyrus showed consistent increases, indicating the brain’s dynamic nature. The observation that connectivity in the bilateral insular cortex initially decreased but later normalized again at follow-up reinforces the idea of the brain’s remarkable plasticity and resilience.

## Discussion

### Main findings

Mission duration significantly impacts the human brain, prompting the inclusion of various brain regions as features in the analyses. Long-duration spaceflight presents a myriad of challenges to human physiological systems. As highlighted by studies (Van Ombergen et al., 2019; McGregor et al., 2023; Roberts et al., 2021; Hupfeld et al., 2020), extended spaceflights lead to pronounced ventricular enlargement. This expansion, although evident after 6 months in space, appears to taper off thereafter. Additionally, longer intervals between missions correlate with a more pronounced post-flight ventricular expansion. It’s worth noting that while ventricular expansion is a hallmark of aging, spaceflight accelerates this process far beyond typical aging rates (Hupfeld et al., 2021). This suggests that the enlargement observed post-flight is not merely a byproduct of brain atrophy. Instead, it is more closely tied to alterations in the CSF dynamics. There’s a prevailing hypothesis suggesting that such disruptions in CSF are the primary driver behind these changes (Kramer et al., 2015). Extended exposure to microgravity might cause the brain to shift upwards, leading to cortical crowding and compression, particularly at the brain’s apex (Lövdén et al., 2010). This could potentially disrupt the CSF flow in the subarachnoid space around the superior cortical regions. Another contributing factor could be the hindrance of CSF resorption into the superior sagittal sinus due to affected arachnoid granulations (Roberts & Petersen, 2019). Essentially, the fluid shifts induced by microgravity might compromise CSF outflow, leading to cerebral venous congestion (Lövdén et al., 2013). This impediment, combined with an increased intracranial CSF volume, is likely responsible for the observed ventricular enlargement (Van Ombergen et al., 2019).

### The effects of intracranial pressure (ICP) rise

Upon entering a microgravity environment, the absence of hydrostatic pressure within the body’s caudocranial fluid columns leads to an immediate cephalic fluid shift and probable onset of SANS, as explained in the introduction section (Nelson et al., 2014). In addition to this syndrome, chronic intracranial hypertension has been increasingly associated with acquired deformities in the pituitary gland (Yuh et al., 2000). Evidence suggests that the majority of astronauts may experience pituitary gland deformation. This deformation is thought to arise from the compounded effect of increased brain and CSF volumes, which aren’t sufficiently counteracted by the decreased blood volume, resulting in elevated ICP (Mase et al., 1998). Furthermore, elevated peak-to-peak velocities in the CSF point to diminished intracranial compliance. This reduction can potentially amplify the pulsatility of ICP related to cerebral perfusion (Eide et al., 2010). Within the confines of a rigid skull, prolonged spaceflight might instigate continual surges in both the average and pulsatile ICP due to the expansion of brain and CSF volumes (Mokri, 2001). Such pressure escalations could detrimentally impact ocular structures and vision (Kramer et al., 2020). Interestingly, while post-spaceflight assessments revealed an association between increased total retinal thickness and lateral ventricle volume, it suggests that CSF distribution changes—potentially responsible for structural alterations in the brain—are not the primary culprits behind optic disc edema (Marshall-Goebel et al., 2021).

### Effects on behavior

The observable morphological changes in the brain due to microgravity conditions are not merely structural phenomena; they seemingly manifest in tangible behavioral impacts. Delving deeper into the relationship between brain activity and behavior allows for a more nuanced understanding of the functional implications of these brain alterations. Drawing from the wider domain of neuroimaging studies, one can discern that behavioral shifts during cognitive tasks—especially those centered on postural adjustments and reaction times—are intrinsically linked to structural changes within the brain (Roberts et al., 2019). One of the most salient examples of this intertwining of brain structure and behavior can be observed in astronauts’ postural control. The caudate nucleus, integral for motor functions, undergoes considerable structural transformation in microgravity environments, potentially leading to discernible postural control deficits in astronauts. Interestingly, similar phenomena have been documented in medical conditions on Earth: idiopathic normal pressure hydrocephalus, a condition characterized by an abnormal gait, is consistently linked with ventricular enlargement in brain scans, implicating the caudate nucleus’s involvement (DeVito et al., 2007). Compared to healthy individuals, patients with this condition often exhibit a reduced caudate nucleus volume coupled with hypometabolism (Townley et al., 2018).

Further emphasizing the critical role of the caudate nucleus in gait regulation, perfusion in this region has been linked to marked improvements in gait in patients diagnosed with idiopathic normal pressure hydrocephalus, particularly post-shunt placement (Ziegelitz et al., 2015). An intriguing observation from our reviewed studies pointed towards a linkage between spaceflight and alterations in the gray matter volume of the paracentral lobule. Such changes might profoundly affect an astronaut’s capacity to recover post-fall. In fact, a thicker preflight paracentral lobule might act as a protective measure, mitigating the adverse effects of spaceflight on motor impairments post-mission (Koppelmans et al., 2022). Moreover, during vestibular stimulations administered pre- and post-spaceflight, substantial variations in activation across a plethora of sensory and motor brain regions were detected. Among these, the precentral gyrus—a region pivotal for motion planning and execution—exhibited a heightened correlation with balance changes from pre to post-flight scenarios (Hupfeld et al., 2022b). This underscores the pivotal role the precentral gyrus assumes in post-flight balance performance, acting as the brain’s command center for the initiation and regulation of movements (Svoboda & Li, 2018).

### Structural gray matter alterations

An intriguing spatial pattern emerges when assessing gray matter volume changes. Observations highlighted an uptick in the gray matter volume in posterior-parietal regions, while fronto-temporal regions showed a decline. Concurrently, the FW content revealed a similar dichotomy: an increase at the base of the cerebellum and a decrease along the posterior vertex (Lee et al., 2021). The simultaneous elevations and depressions in FW are indicative of cranial fluid redistributions. Especially compelling is the observation of ventral pooling of FW, which aligns with the thesis that the brain undergoes a global upward shift. This redistribution, accompanied by a concurrent decrease in FW in posterior-parietal areas, gives credence to the idea that the vertex’s brain parenchymal crowding is potentially responsible for the augmented gray matter volume in these regions.

### Correlations with age

The upward displacement of the brain has not only been a singular observation but has been consistently noted across multiple astronaut studies (Van Ombergen et al., 2018; Lee et al., 2019). The dynamics of white matter volume changes due to spaceflight appear to be independent of an astronaut’s age. There’s evidence of a recognized quadratic relationship between white matter volume and age, unaffected by gender (Hasan et al., 2007). This volume expands during one’s childhood, peaks between the third and fourth decades, and subsequently wanes. Taking into account the average age of astronauts, it becomes evident that the observed alterations in white matter volume aren’t age-mediated (Lee et al., 2021).

### Structural white matter alterations

Moreover, post-spaceflight assessments have shown significant drops in FA and AD values in astronauts, coupled with a surge in RD values. These markers suggest potential structural disarray across multiple white matter tracts (Lee et al., 2019). These changes can affect a variety of brain regions, especially regions that process visuomotor control and sophisticated visuospatial processing (Rodríguez-Herreros et al., 2015). Specifically, the SLF plays a pivotal role in synchronizing body posture and spatially directed actions by bridging the temporoparietal with the prefrontal cortices (Spena et al., 2006). Notably, astronauts manifesting the most pronounced balance disturbances showed reduced AD within the SLF, pointing to diminished axonal density and structure (Arfanakis et al., 2002). Additionally, MD values swelled within the right occipital cortex, especially pronounced in the right fusiform gyrus, an area intricately linked with neural circuits governing recognition (Riascos et al., 2019). These changes in regional gray and white matter that have close ties to visual function, particularly within the occipital cortex, thalami, and posterior thalamic radiations, might be suggestive of cerebral edema and fluid accumulation along the visual pathway. This accumulation results from microgravity-induced intracranial hypertension during extended space missions (Bryan, 2017). There’s also a supposition that optic nerve changes might have a domino effect, culminating in the degradation of the optic radiation and the associated visual cortices.

### Effects of prior spaceflight experiences

A differential response to spaceflight based on experience levels has been brought to the fore by the variances in FW distribution in astronauts. Specifically, astronauts with a more extensive mission portfolio exhibited decreases in FW within the anterior and medial regions of the brain, including the lateral ventricle. In contrast, those newer to spaceflight presented with FW increases in these regions (McGregor et al., 2023; Lee et al., 2019). This dichotomy gives rise to intriguing hypotheses regarding the cumulative impacts of spaceflights. One potential explanation posits that the cumulative impacts of multiple missions render the brains of seasoned astronauts less compliant. The brain’s response, following numerous exposures to space and the associated recovery periods, might change.

Within this framework, the ventricles of these experienced astronauts might be less pliable, limiting their ability to expand. Such an inability to expand compromises the ventricles’ function as overflow zones, potentially resulting in reduced CSF resorption (Roberts et al., 2021; Roberts & Petersen, 2019). This could signify that repeated exposures to microgravity environments cause long-term structural changes in the brain, possibly due to reduced adaptive capabilities over time. In contrast, astronauts with fewer missions, often referred to as novices or less-experienced flyers, might possess a more compliant brain. Their neural environment may more readily accommodate the cranial fluid shifts characteristic of spaceflight, allowing for ventricular expansion. This adaptability might be emblematic of the brain’s initial responses to the challenges presented by a microgravity environment. Thus, the gravitational transitions experienced during spaceflight seem to exert a profound influence on the FW compartment. The brain’s inherent plasticity and overall structural framework might undergo transformations when subjected to recurring gravitational transitions. This process could involve a repeated recalibration of the brain’s structure and fluid dynamics in response to varying gravitational pressures, suggesting that the duration and frequency of spaceflights might also affect the brain.

### Functional alterations

Task-based and resting-state connectivity assessments provide unique yet interconnected insights into brain functionality. Task-based connectivity offers a snapshot of the functional brain’s real-time engagement during specific motor actions. Resting-state connectivity, on the other hand, sheds light on the inherent activity patterns of extensive neural networks, typically in the absence of overt tasks or stimuli (Heine et al., 2012). Following spaceflight, resting-state connectivity can reveal diminished and enhanced network activities in response to microgravity. This juxtaposition of connectivity data implies the neuroplastic changes within the motor system, shedding light on how the brain remodels itself in response to varying environmental demands. Some of these adaptations are indeed reflected in post-flight alterations in task-based connectivity within somatosensory domains. Notably, post-flight evaluations identified diminished connectivity within the somatosensory cortex – a pivotal hub in the superior parietal cortex that receives multisensory inputs – during tactile processing tasks (Salazar et al., 2023; Pechenkova et al., 2019). This diminishment in connectivity was not restricted to specific tasks. The lateral superior occipital gyrus similarly exhibited subdued resting-state functional connectivity with other brain regions, underscoring the possibility of a broader sensory integration compromise within the brain post-flight (McGregor et al., 2021).

A striking observation was the alterations in visual cortical connectivity following spaceflight. While task-based connectivity studies showed interactivity patterns of the visual cortex, especially with frontal regions, the resting-state connectivity pinpointed shifts in the primary visual cortex interaction dynamics with other brain regions. The implications of these nuanced changes in visual cortical connectivity, especially in the context of spaceflight, necessitate comprehensive exploration. There is reason to hypothesize that these changes are aligned with shifts in vestibular feedback. Alterations in the vestibular inputs during flight and the prioritization of visual and proprioceptive inputs over vestibular ones could be the driving factors behind these connectivity shifts (Hupfeld et al., 2022b). As we advance, understanding the genesis and ramifications of these neural connectivity adaptations will be imperative for safeguarding astronaut well-being.

### Possible roles of neuroplasticity

A salient aspect of the presented findings emerges from the combination use of quantitative MRI and DTI-based analysis. One key revelation pertains to the intriguing interplay of age with the insula’s morphology. Despite age-related thinning of the insula, a compensatory phenomenon was observed where its surface area experienced a slight augmentation, preventing any net volume loss (Hasan et al., 2018). Such an observation is not merely an anatomical anomaly but suggests profound implications regarding the neuroplastic capabilities of astronauts’ brains. This expansion in the surface area, despite thinning, hints at underlying mechanisms of neuroplasticity or perhaps a form of compensatory neurogenesis specific to this occupational cohort. It is plausible that the unique physiological demands and environmental adaptations induce specific brain remodeling patterns, which deviate from typical age-related neurodegenerative trajectories observed in the general populace (Storsve et al., 2014).

Essentially, the astronaut brain, when subjected to the rigors of space, might be eliciting a protective or adaptive response, invoking neuroplastic mechanisms to lessen normative age-induced volume losses. This hypothesis, if further substantiated, could have profound ramifications. Not only does it shed light on the brain’s inherent resilience and adaptability, but it also underscores the need to reconceptualize our understanding of the aging brain, especially within contexts as unique as spaceflight. The challenge ahead lies in further delineating these compensatory mechanisms, identifying potential triggers, and exploring whether such adaptations offer any long-term neuroprotective benefits or if they come with their own set of trade-offs.

### Findings of other reviews

The findings of our study align with those of (Roy-O’Reilly et al., 2021), as they reported macrostructural changes in the brain position, tissue volumes, and CSF dynamics following spaceflights. In their review, they have also implied microstructural alterations in regions related to vestibular, cerebellar, visual, motor, somatosensory, and cognitive functions. However, these changes may not merely result from physical stressors in spaceflight. In another review by (Yin et al., 2023), microgravity, along with isolation, confinement, noise, and circadian rhythm disturbances, was reported to cause depression and cognitive decline. The burden of spaceflights on the mental health of astronauts is of great significance (Arone et al., 2021), and whether these cognitive or psychiatric symptoms cause or are caused by microstructural and macrostructural brain changes remains to be elucidated.

### Limitations

Our review is not without limitations. One limitation is the relatively small sample size of the astronauts included in the studies. Small sample sizes can reduce the statistical power of the study and subsequently decrease the sensitivity to detect true differences and increase the likelihood of observing a false positive result (Serdar et al., 2021). Another point of contention arises from the diverse spacefaring experience of the subjects. As many space travelers embark on multiple missions throughout their careers, the data becomes a heterogeneous blend of physiological and neurological responses from both rookie and veteran astronauts. This heterogeneity complicates the interpretation of findings, as repeated exposures to microgravity might induce different neural responses compared to initial exposures. Moreover, there is an intrinsic variability across the studies regarding the MRI modalities. The disparate acquisitions spanning Structural MRI, dMRI, resting-state fMRI, and task-based fMRI, paired with the focus on different brain regions, may introduce discrepancies in the results. Such heterogeneity simultaneously dilutes the ability to draw unified conclusions.

### Practical significance

Yet, even within the constraints of these limitations, the core message remains unambiguous: spaceflight induces tangible alterations in brain structure. This assertion amplifies the urgency for a deeper and more systematic inquiry into the adaptive mechanisms of the human brain in response to prolonged spaceflight. The consequences of such research extend beyond academic interest, reaching into the realms of safety protocols for the crew aboard ISS, and more critically, for those participating in missions to extraterrestrial destinations like the moon and Mars. Evidently, due to heightened interplanetary aspirations, there is an evident lacuna in our understanding of the neuroanatomical and physiological implications of such endeavors. Our analysis underscores the need for a holistic approach, entailing advanced neuroimaging protocols and rigorous long-term follow-up studies on astronauts. A comprehensive understanding of the relationship between gravitational changes and CSF homeostasis is not just crucial for grasping the intricacies of space-induced physiological changes; it can provide a broader comprehension of pathologies like idiopathic normal pressure hydrocephalus that afflict populations even on Earth.

### Future directions

More studies are needed to better shed light on the specific effects of spaceflights on the brains of astronauts. These could include studies recruiting larger sample sizes and longitudinal assessments while controlling for the perceived physical stressors, psychological stressors, prior experiences, the length of the travel, and the destination. Moreover, complementary studies with other imaging tools, such as Soma and Neurite Density Imaging (SANDI), Neurite Orientation Dispersion and Density Imaging (NODDI), and fMRI studies assessing both resting state and task-related connectivity can also help to address the current knowledge gap in this regard. Finally, the included studies were limited to the imaging modalities conducted on Earth after and/or before the spaceflights. In the near future, there might be feasible neuroimaging tools to conduct studies in the ISS itself, as there are already grants to build ankle-sized MRI scanners to assess the bone health of the astronauts (University of Saskatchewan, 2018).

## Conclusions

Spaceflight induces tangible alterations in brain structure. The unique physiological demands and environmental adaptations required during space missions lead to specific brain remodeling patterns that deviate from typical age-related neurodegenerative trajectories observed in the general population. Furthermore, different brain regions and repeated exposures to microgravity may induce different neural responses. The absence of hydrostatic pressure in a microgravity environment leads to an immediate cephalic fluid shift, resulting in increased intracranial CSF volume. Despite all these findings, neuroimaging assessments might not reflect all the impact of these new environmental stressors during a spaceflight. Therefore, other complementary tests assessing hormonal, immunological, glial, and also psychological profiles are needed to improve our understanding of spaceflight effects on astronauts.
